# Emergence of Algal Blooms: The Effects of Short-Term Variability in Water Quality on Phytoplankton Abundance, Diversity, and Community Composition in a Tidal Estuary

**DOI:** 10.3390/microorganisms2010033

**Published:** 2014-01-08

**Authors:** Todd A. Egerton, Ryan E. Morse, Harold G. Marshall, Margaret R. Mulholland

**Affiliations:** 1 Department of Biological Sciences, Old Dominion University, Norfolk, VA 23529, USA; E-Mail: hmarshal@odu.edu; 2 Graduate School of Oceanography, The University of Rhode Island, Narragansett, RI 02882, USA; E-Mail: morse@mail.uri.edu; 3 Department of Ocean, Earth and Atmospheric Sciences, Old Dominion University, Norfolk, VA 23529, USA; E-Mail: mmulholl@odu.edu

**Keywords:** algal blooms, cryptomonads, dinoflagellates, Chesapeake Bay, *Gymnodinim instriatum*

## Abstract

Algal blooms are dynamic phenomena, often attributed to environmental parameters that vary on short timescales (e.g., hours to days). Phytoplankton monitoring programs are largely designed to examine long-term trends and interannual variability. In order to better understand and evaluate the relationships between water quality variables and the genesis of algal blooms, daily samples were collected over a 34 day period in the eutrophic Lafayette River, a tidal tributary within Chesapeake Bay’s estuarine complex, during spring 2006. During this period two distinct algal blooms occurred; the first was a cryptomonad bloom and this was followed by a bloom of the mixotrophic dinoflagellate, *Gymnodinium instriatum*. Chlorophyll *a*, nutrient concentrations, and physical and chemical parameters were measured daily along with phytoplankton abundance and community composition. While 65 phytoplankton species from eight major taxonomic groups were identified in samples and total micro- and nano-phytoplankton cell densities ranged from 5.8 × 10^6^ to 7.8 × 10^7^ cells L^−1^, during blooms, cryptomonads and *G. instriatum* were 91.6% and 99.0%, respectively, of the total phytoplankton biomass during blooms. The cryptomonad bloom developed following a period of rainfall and concomitant increases in inorganic nitrogen concentrations. Nitrate, nitrite and ammonium concentrations 0 to 5 days prior were positively lag-correlated with cryptomonad abundance. In contrast, the *G. insriatum* bloom developed during periods of low dissolved nitrogen concentrations and their abundance was negatively correlated with inorganic nitrogen concentrations.

## 1. Introduction

In estuarine systems, phytoplankton communities are highly variable, affected by numerous environmental and ecological factors including water temperature, salinity, light intensity, nutrient availability, inter- and intra-specific competition among the algae, and predation [1,2,3,4]. Many of these environmental factors vary on short time scales in estuaries due to tidal and diel fluctuations in physical/chemical parameters and episodic nutrient inputs from precipitation events [5,6,7,8]. Because of their short generation times, phytoplankton populations can respond rapidly to environmental and ecological forcing [9,10,11] Consequently, changes in algal community composition and diversity can occur over relatively short time periods in response to environmental variability [12,13,14] and this can affect ecological function [15,16].

Algal blooms usually involve rapid changes in phytoplankton community composition, where in phytoplankton communities become dominated by a single (or a few) species over the course of days, resulting in nearly monospecific assemblages that can then persist for weeks to months [17,18]. Such monospecific algal blooms appear to be increasing in frequency and magnitude, and nutrient over-enrichment has been implicated as a causal factor [19,20]. However, linking blooms to a proximate trigger has proven difficult because blooms are generally sampled only after they become visible when cell densities are already high enough to discolor water. As a result, the environmental conditions during bloom initiation are usually unknown. In addition, bloom organisms can be transported from sites of initiation to the areas where algal biomass accumulates and blooms are observed [21].

Because environmental conditions and phytoplankton communities can change rapidly in estuaries due to physical and meteorological forcing, monthly monitoring is not sufficient to document bloom initiation in response to short-term environmental variability. Recent studies aimed at identifying causal factors promoting bloom formation sampled more frequently and demonstrated that rapid changes in algal biomass as blooms developed and then dissipated associated with short-term variability in water quality [7,8,22,23]. Continuous monitoring and high resolution mapping has further highlighted the rapid fluctuations and spatial variability in nutrient and dissolved oxygen concentrations, chlorophyll biomass, temperature and salinity over diurnal timescales [7,21,24]. Daily sampling studies have also been used to document the relationships between water quality parameters and algal community composition that occur over short periods of time [7,12,25]. These studies suggested that meteorological forcing was important in driving changes in algal community structure.

The objectives of this study were to identify short-term changes in phytoplankton species composition and diversity and relate these with water quality parameters and meteorological forcing, the development of mono-specific blooms, and algal species diversity in a tidal estuarine system during spring, when rainfall is usually frequent and the algal community diverse.

## 2. Experimental Section

### 2.1. Study Site

The Lafayette River, located in Norfolk, VA, USA, is a tributary of the Elizabeth River that flows into the lower James River near its confluence with the Chesapeake Bay. It is a tidal river, approximately 8 km in length, with a mean depth of 1.3 m, and a maximum channel depth of 7.6 m [26]. The river is surrounded by residential and commercial development, within an urban watershed of 43.28 km^2^, and a shoreline that includes bulk headed regions, marinas, private docks and wetland marshes of *Spartina alterniflora* [26,27,28,29]. Freshwater input is delivered through precipitation and shoreline drainage that includes 13 storm sewers and overflow drains [27,30]. Seasonal dinoflagellate blooms common in this river include: *Prorocentrum minimum* (early spring) and *Akashiwo sanguinea* and *Cochlodinium polykrikoides* (summer and fall) [22,31,32,33]. The river has been identified as an initiation site for regional dinoflagellate blooms dominated by *C. polykrikoides* during summer and fall [20].

### 2.2. Methods and Materials

Surface water samples were collected once a day during the incoming tide, approximately 2 h after low tide, from a stationary floating dock on the Lafayette River between 20 April and 25 May 2006, as described by Morse *et al*. [7]. The mean water depth at the station was 0.9 m. Water temperature, salinity, pH, and dissolved oxygen were measured daily just before collecting water samples using a Hydrolab Data Sonde 4a water quality multiprobe (Hach Company, Loveland, CO, USA). Rainfall and air temperature were recorded at Norfolk International Airport, <10 km from the Lafayette River station.

Samples (25–50 mL) were collected onto Whatman GF/F filters (pore size ~0.7 μm) and frozen for later analysis of chlorophyll *a* (Chl*a*). Chl*a* was measured fluorometrically after extraction in acetone within 2 weeks of sample collection [34]. Samples were filtered through 0.2 μm Supor filters and the filtrate frozen for later analysis of dissolved nutrient concentrations. Dissolved nitrate, nitrite, urea, phosphate, and silicate were measured colorimetrically using an Astoria Pacific nutrient autoanalyzer according to the manufacturer’s specifications. Ammonium was analyzed colorimetrically using the phenolhypochlorite method [35].

Nano- and microphytoplankton samples (500 mL) were collected from the surface (<1 m), preserved with Lugol’s solution (1% final concentration), and quantified using an inverted microscope (Nikon TS100) at 150–600× magnification following a modified Utermöhl settling and siphoning protocol [36]. Autotrophic picoplankton samples, collected at the same time and depth were preserved with gluteraldehyde (2%) and quantified using epifluorescence microscopy (Nikon E600) at 1000× magnification [37]. Phytoplankton cell volume was calculated based on observed cell dimensions and phytoplankton carbon (C) biomass calculated using established biovolume to biomass relationships [38]. Dinoflagellate species identities were positively confirmed using scanning electron microscopy (SEM). Samples for SEM were fixed with gluteraldehyde and osmium tetroxide, dehydrated through an ethanol series, dried using a critical point drier, sputter-coated with gold-paladium, and analyzed using a LEO 435VP (LEO Electron Microscopy Ltd., Thornwood, NY, USA) [39]. Phytoplankton diversity was calculated daily using both species richness (number of species per sample) and the Shannon index (*H′*; Equation (1)), the latter incorporates the relative abundance of each species and therefore is commonly used as a measure of species evenness [40].



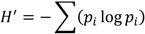
(1)
*p_i_* is the proportion of the total algal biomass of species *i.*

The daily sampling regimen was designed to measure phytoplankton species abundance and nutrient concentrations prior to, during, and following algal blooms. Phytoplankton species abundance and diversity were compared with corresponding environmental data using Pearson correlation analysis. Because algal growth rates are on the order of days, we anticipated a lag response of phytoplankton abundance relative to nutrient concentrations and associated meteorological forcing [7]. The lag correlation analyses conducted here compared nutrient concentrations at one day intervals over an 11 day window encompassing the period before and after observed blooms of a cryptomonad and the dinoflagellate, *Gymnodinium instriatum*. Because biological interactions such as competition and predation are known to influence phytoplankton composition, we also used a lag correlation analysis of species richness, *H′*, and the abundance of other dominant phytoplankton groups relative to dinoflagellate and cryptomonad abundance.

Regression analysis was used to examine the relationship between species diversity (both richness and *H′*) and total algal biomass. These results were compared to regression analyses of phytoplankton diversity and biomass data collected by the authors from nearby sites during the same time period as part of the Virginia Chesapeake Bay monitoring program (*n* = 26). Because previous studies identified both linear and non-linear (unimodal) relationships between the variables (e.g., [41]), analysis of variance was conducted to test for significant linear and quadratic relationships using regression models (SPSS 20; IBM). If both regression models were significant for a particular analysis, a partial *F* test was used to determine if the quadratic model significantly improved the explanation of the data relative to the linear model [42,43].

## 3. Results and Discussion

### 3.1. Meteorological and Physical Parameters

**Figure 1:**
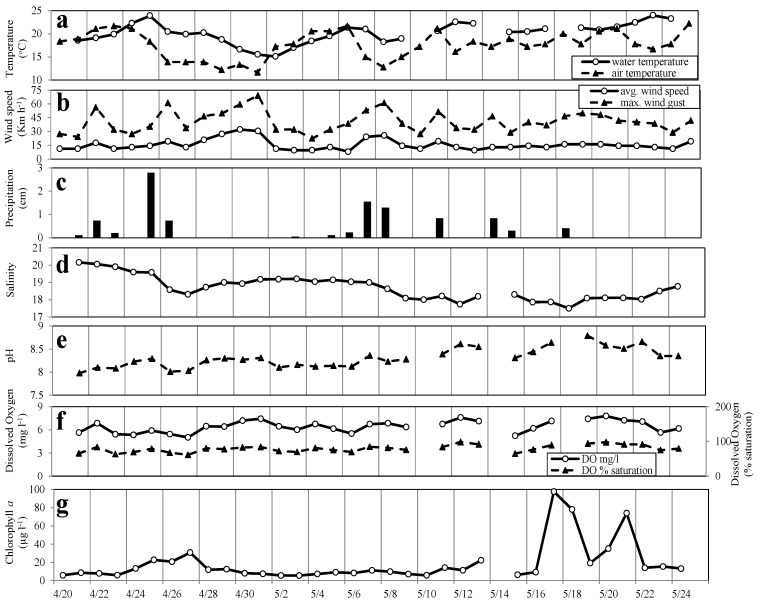
Daily measurements of physical and chemical parameters in the Lafayette River from 20 April to 25 May 2006. Water temperature (°C) was measured at the sampling site using the Hydrolab and mean daily air temperatures were measured at Norfolk International Airport (ORF) (**a**). Mean daily wind speed and maximum daily speed of wind gusts (miles h^−1^) were measured at Norfolk International Airport (ORF) (**b**). Daily cumulative precipitation (cm) was also measured at ORF (**c**). Salinity (**d**), pH (**e**), dissolved oxygen (mg L^−1^), and percent saturation (**f**) were measured using the Hydrolab and chlorophyll *a* measurements (µg L^−1^) were made daily on surface water samples (**g**).

Over the 34-day sampling period, mean daily air temperatures ranged from 11.7 to 21.7 °C, and water temperatures ranged from 15.1 to 24.0 °C (Figure 1a). Average daily wind speeds were variable and ranged from 8 to 32 km h^−1^ with gusts exceeding 48 km h^−1^ (30 miles h^−1^) on 9 days; maximum wind gusts of 69 km h^−1^ were observed on May 1 (Figure 1b). During the sampling period there were 8 rain events recording 0.5 cm or more of precipitation (Figure 1c). Salinity at the sampling site decreased over the sampling period, with a maximum of 20.2 observed on 20 April and a minimum of 17.5 on 18 May. Salinity decreased following periods of rainfall (Figure 1d). The average pH at our study site was 8.31, but pH ranged from 7.98 to 8.79 (Figure 1e). Dissolved oxygen concentrations ranged from 5.0 to 7.8 mg L^−1^; this was 61.6% to 98.1% saturation (Figure 1f).

### 3.2. Phytoplankton Abundance, Composition and Diversity

**Figure 2:**
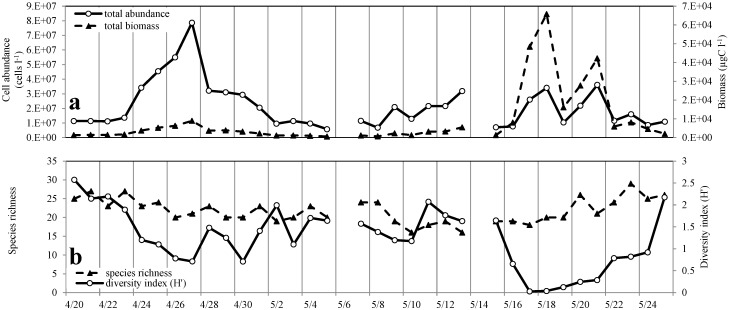
Nano and micro phytoplankton abundance and biomass (**a**), and phytoplankton diversity (Shannon diversity index *H′*) and species richness (**b**) in daily samples collected from the Lafayette River between 20 April and 25 May 2006.

Chlorophyll *a* (Chl*a*) concentrations ranged from 5.54 to 97.6 µg L^−1^ over the 34-day study period, but were less than 20 µg L^−1^ for all but 8 of the days (Figure 1g). Elevated Chl*a* concentrations, 20.8–30.7 µg L^−1^, observed between 24 April and 1 May, were associated with a cryptomonad bloom (Figure 1g). High Chl*a* concentrations observed between 16 and 24 May (35.2–97.6 µg L^−1^) were associated with high abundances of the dinoflagellate *Gymnodinium instriatum* (Figure 1g). Nano- and microphytoplankton cell densities ranged from 5.8 × 10^6^ to 7.8 × 10^7^ cells L^−1^ throughout the study period (Figure 2a); picoplankton abundances ranged from 3.7 × 10^6^ to 1.3 × 10^9^ cells L^−1^ (data not shown).

While dominated by a single species during blooms, the phytoplankton community consisted of 65 taxa from 8 major taxonomic groups, with 41 taxa present on 5 or more days (Table 1). There were 37 species of diatoms, 17 dinoflagellate species, 3 cyanobacteria, 2 silicoflagellates, 2 chlorophytes, and 1 each of cryptomonads, euglenophytes and prasinophytes. While diatoms were the most diverse group, consisting of mainly centric species (e.g., *Skeletonema costatum* and *Chaetoceros* spp.), they never represented more than 49% of the total phytoplankton present, and were generally much less abundant than the phytoflagellates.

**Table 1:** Summary statistics of phytoplankton abundance data (cells L^−1^) of the 41 taxa observed in the Lafayette River samples at least 5 times during the study. The two bloom taxa are identified in bold.

Phytoplankton Taxa	Abundance		
	Mean Value	Minimum Value	Maximum Value
**Diatoms**			
unidentified Centrales 10–30 µm	2.0 × 10^6^	1.0 × 10^3^	5.3 × 10^6^
unidentified Centrales 30–60 µm	1.0 × 10^4^	2.6 × 10^2^	1.1 × 10^5^
*Chaetoceros pendulus*	6.7 × 10^2^	2.6 × 10^2^	1.0 × 10^3^
*Chaetoceros* sp.	1.2 × 10^5^	7.7 × 10^2^	4.3 × 10^5^
*Cocconeis* sp.	2.8 × 10^2^	2.6 × 10^2^	5.1 × 10^2^
*Coscinodiscus* sp.	5.7 × 10^2^	2.6 × 10^2^	1.3 × 10^3^
*Cyclotella* sp.	1.1 × 10^5^	5.1 × 10^2^	4.3 × 10^5^
*Cylindrotheca closterium*	5.3 × 10^2^	2.6 × 10^2^	1.5 × 10^3^
*Dactyliosolen fragilissimus*	2.9 × 10^4^	5.1 × 10^2^	3.2 × 10^5^
*Gyrosigma fasciola*	3.1 × 10^2^	2.6 × 10^2^	5.1 × 10^2^
*Leptocylindrus minimus*	7.7 × 10^4^	7.7 × 10^2^	5.4 × 10^5^
*Navicula* sp.	7.3 × 10^2^	2.6 × 10^2^	3.8 × 10^3^
*Nitzchia* sp.	2.6 × 10^2^	2.6 × 10^2^	2.6 × 10^2^
unidentified Pennales 10–30 µm	2.6 × 10^5^	2.6 × 10^2^	8.7 × 10^5^
unidentified Pennales30–60 µm	8.9 × 10^3^	2.6 × 10^2^	1.1 × 10^5^
unidentified Pennales > 60 µm	9.0 × 10^2^	2.6 × 10^2^	2.8 × 10^3^
*Pleurosigma* sp.	2.6 × 10^2^	2.6 × 10^2^	2.6 × 10^2^
*Rhizosolenia setigera*	1.2 × 10^3^	2.6 × 10^2^	3.8 × 10^3^
*Skeletonema costatum*	1.0 × 10^5^	1.0 × 10^3^	9.7 × 10^5^
*Thalassiosira* sp.	6.7 × 10^2^	2.6 × 10^2^	1.3 × 10^3^
**Dinoflagellates**			
*Akashwio sanguinea*	3.0 × 10^3^	2.6 × 10^2^	1.8 × 10^4^
*Cochlodinium polykrikoides*	1.1 × 10^4^	5.1 × 10^2^	3.7 × 10^4^
unidentified dinoflagellate	1.8 × 10^5^	5.1 × 10^2^	5.4 × 10^5^
*Dinophysis punctata*	5.4 × 10^2^	2.6 × 10^2^	2.0 × 10^3^
*Diplopsalis lenticula*	3.1 × 10^2^	2.6 × 10^2^	5.1 × 10^2^
*Gymnodinium* sp.	8.9 × 10^4^	2.6 × 10^2^	8.7 × 10^5^
***Gymnodinium instriatum***	**3.7 × 10^6^**	**2.6 × 10^2^**	**3.1 × 10^7^**
*Heterocapsaro tundata*	6.6 × 10^5^	1.1 × 10^5^	3.9 × 10^6^
*Heterocapsa triquetra*	1.0 × 10^4^	2.6 × 10^2^	1.1 × 10^5^
*Polykrikos kofoidii*	5.9 × 10^3^	1.0 × 10^3^	4.5 × 10^4^
*Prorocentrum micans*	9.3 × 10^2^	2.6 × 10^2^	5.9 × 10^3^
*Prorocentrum minimum*	2.2 × 10^4^	2.6 × 10^2^	4.3 × 10^5^
*Protoperidinium* sp.	7.5 × 10^2^	2.6 × 10^2^	1.8 × 10^3^
*Scrippsiella trochoidea*	7.3 × 10^2^	2.6 × 10^2^	2.3 × 10^3^
**Cryptomonads**			
***Cryptomonas* sp.**	**1.5 × 10^7^**	**5.4 × 10^5^**	**7.6 × 10^7^**
**Cyanobacteria**			
*Lyngbya* sp.	5.3 × 10^5^	5.1 × 10^2^	2.3 × 10^6^
**Chlorophytes**			
*Ankistrodesmus falcatus*	8.7 × 10^3^	2.6 × 10^2^	1.1 × 10^5^
*Chlamydomonas* sp.	3.6 × 10^5^	1.1 × 10^5^	8.7 × 10^5^
**Euglenoids**			
*Euglena* sp.	7.6 × 10^4^	2.6 × 10^2^	4.3 × 10^5^
*Eutreptia lanowii*	2.0 × 10^3^	5.1 × 10^2^	5.6 × 10^3^
**Prasinophytes**			
*Pyramimonas* sp.	7.2 × 10^4^	2.6 × 10^2^	3.2 × 10^5^

**Figure 3:**
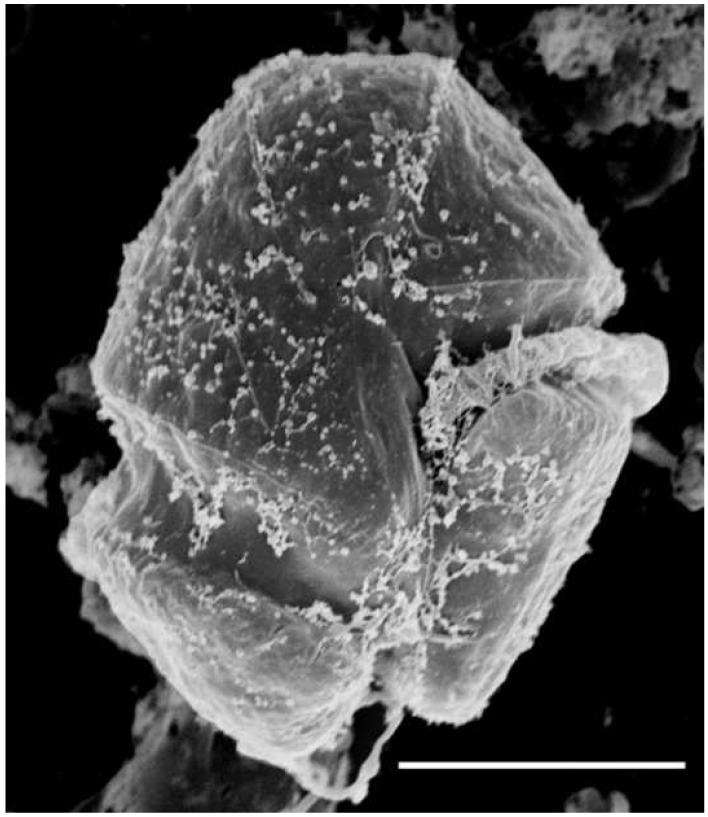
Scanning electron micrograph of a *Gymnodinium instriatum* vegetative cell, collected at the study site on 18 May 2006, during a bloom of this dinoflagellate. Scale bar = 10 μm.

Cryptomonad taxonomic identification is notoriously problematic due to the cells’ sensitivity to chemical fixatives and the small number of morphological features that distinguish them from one another [44,45]. The morphology and size of the cryptomonads appeared consistent throughout the course of the study. The cells were comma-shaped, with a round anterior and a reflex curved pointed antapex with an average length of 18.3 μm and an average maximum width of 8.3 μm. While consistent morphological features were observed during the sampling period, the cryptomonad bloom was conservatively identified as *Cryptomonas* spp., indicating the possible presence of multiple species. *Gymnodinium instriatum* was recognized by its morphological features including the displacement of the cingulum and the shape of the apical groove (Figure 3) [46] and identified using the most recent nomenclature [47]. 

**Figure 4:**
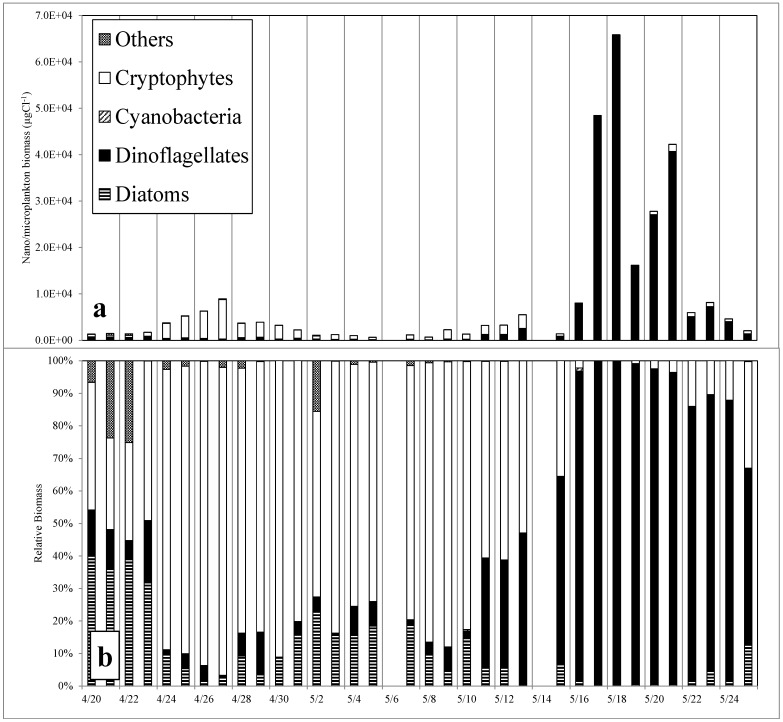
Biomass (µg C L^−1^) of the major taxonomic groups within the Lafayette River from 20 April to 25 May shown as absolute algal biomass for each major taxonomic group (**a**) and each taxonomic group as a percentage of the total algal biomass (**b**).

Phytoflagellates, specifically cryptomonads and dinoflagellates, were the dominant algae throughout the study. At the beginning of the study period in April, the algal community was dominated by cryptophytes and diatoms but also contained substantial populations of dinoflagellates and other species. The most abundant taxon was *Cryptomonas* spp., which reached a maximum density of 7.7 × 10^7^ cells L^−1^ on 27 April. At its peak, this group represented 96.1% of the total phytoplankton abundance and 91.6% of the phytoplankton biomass (Figure 4b). *Cryptomonas* spp. concentrations decreased to 4.0 × 10^6^ cells L^−1^ on May 5 and then increased again having a second smaller peak in abundance of 2.6 × 10^7^ cells L^−1^ on May 13. As the *Cryptomonas* spp. abundance declined, the densities of *Gymnodinium instriatum* rose dramatically beginning May 15. *G. instriatum* reached a maximum density of 3.0 × 10^7^ cells L^−1^ on May 18 (Figure 4a). This represented 89.8% of the phytoplankton abundance and 99.0% of the total phytoplankton biomass (Figure 4b). *G. instriatum* abundance and chlorophyll *a* concentrations decreased on May 19 following a rainfall event (Figure 1c) and then increased again to 1.9 × 10^7^ cells L^−1^ on May 21.

Estimates of phytoplankton biomass made using cell abundance and biovolume were highly correlated with Chl*a* concentrations (*r* = 0.95, *p* = 0.000). Calculated nano-and microphytoplankton biomass ranged from 609 to 65,819 µg C L^−1^, with the highest biomass measured during the *Gymnodinium* bloom from May 16 to 24 (Figure 4a). Picoplankton always contributed less than 1% of total phytoplankton biomass throughout the study ranging from 0.5 to 181 μg C L^−1^ throughout the study.

**Figure 5:**
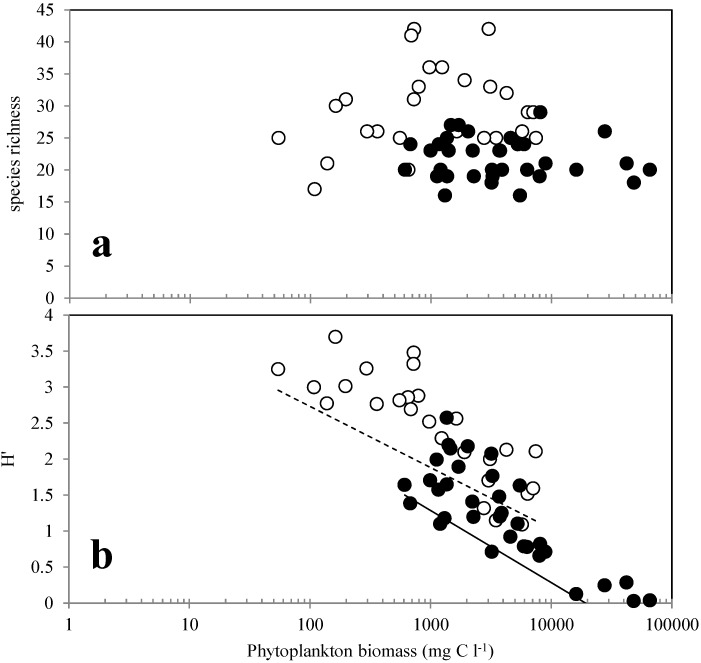
Scatterplots of phytoplankton biomass and phytoplankton diversity expressed as species richness (**a**) and Shannon diversity index *H′* (**b**). Black circles represent measurements of biomass and diversity recorded daily from samples collected in the Lafayette River from 20 April to 25 May 2006. White circles represent algal biomass and diversity measurements recorded at 14 Chesapeake Bay Program Monitoring stations in Virginia during April and May 2006. There was a significant negative linear relationship between species diversity and phytoplankton biomass for both datasets (*p* < 0.0001) (**b**). The solid line shows the relationship for the Lafayette River samples and the dashed line shows the relationship for the Chesapeake Bay Program data.

Species richness was low in the Lafayette River during this study, ranging from 16 to 32 with a mean of 21 taxa identified per sample compared to an average of 32 taxa identified in samples collected from the nearby Chesapeake Bay Monitoring Program station located in the Elizabeth River (SBE5) during the same time period. The Shannon diversity index (*H′*), which includes a measure of species evenness, ranged between 0.03 and 2.57 (Figure 2b) and was lowest during the *Cryptomonas* spp. and *G. instriatum* blooms when these species dominated the phytoplankton populations. However, even when *Cryptomonas* spp. and *G. instriatum* were at their maximum abundance and represented 96.1% and 99.0% of the biomass, respectively, there were still about 20 other phytoplankton species present and species richness did not vary during bloom and non-bloom periods. Diversity rapidly increased again after blooms dissipated (Figure 2b) and there was a significant negative linear relationship between phytoplankton biomass and diversity (*H′*) over the 34 day study (*R*^2^ = 0.637, *p* < 0.0001) (Figure 5a). A negative relationship between phytoplankton biomass and species diversity was also observed during the same time period at other locations within the lower Chesapeake Bay. No significant relationship was observed between species richness and phytoplankton biomass (*p* > 0.05) (Figure 5b).

### 3.3. Nutrient Concentrations

**Figure 6:**
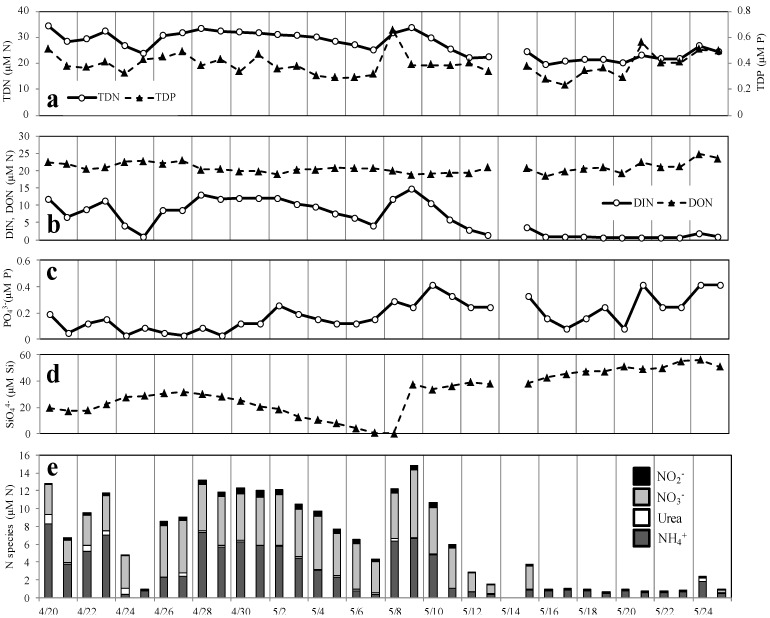
Nutrient concentrations measured in the Lafayette River from 20 April to 25 May 2006. Daily measurements of total dissolved nitrogen (TDN, µM N) and total dissolved phosphorus (TDP, µM P) (**a**), dissolved inorganic nitrogen (DIN, µM N) and dissolved organic nitrogen (DON, µM N) (**b**), orthophosphate (µM P) (**c**), dissolved silicate (µM Si) (**d**), and dissolved nitrogen concentrations by species (**e**).

Dissolved inorganic nitrogen concentrations (DIN) (nitrite, nitrate, and ammonium) ranged from 0.54 to 14.7 µM during the study period. DIN concentrations were highest at the start of the study and lowest after May 16 when dinoflagellate abundances were highest (Figure 6b). While nitrate and ammonium were both abundant during the early part of the study period, after May 15, about the time of the dinoflagellate bloom, nitrate concentrations were depleted and only ammonium was detected.

Nitrate concentrations ranged from the detection limit (0.05 µM) during and after the *Gymnodinium* (May 16 to May 24) bloom to 7.6 µM during the period between the two blooms, and represented a large portion of the available DIN, averaging 41% of the DIN pool over the study period (range was detection limit to 88% of DIN) (Figure 6e). NO_3_^−^ was generally present at high concentrations (>1 μM) until May 16 and then NO_3_^−^ was depleted in the days leading up to the dinoflagellate bloom, during the latter part of the study period. NO_3_^−^ concentrations remained low subsequent to the *G. instriatum* bloom. NO_2_^−^ concentrations were always <1 μM and accounted for less than 10% of the DIN pool throughout the study; the maximum NO_2_^−^ concentration was 0.81 µM (Figure 6e). As with NO_3_^−^, concentrations of NO_2_^−^ were highest following the *Cryptomonas* spp. bloom (30 April to 4 May), and below the detection limit (0.02 µM) during and after the dinoflagellate bloom (May 16 to 24).

Ammonium concentrations were also variable over the study period ranging from 0.4 to 8.3 µM, but were always detectable (>0.02 µM). NH_4_^+^ was the dominant form of DIN during most of the study; concentrations were highest during the first half of the study and 1–2 days following two major precipitation events (25–26 April and 7–8 May). NH_4_^+^ concentrations decreased at the end of the study (May 11 to May 25) and were uniformly low, but detectable, before and during the dinoflagellate bloom. Like NO_2_^−^, urea concentrations were uniformly low (mean concentration of 0.18 µM) throughout the study period and at or below the analytical detection limit (0.05 µM) on 13 of the last 14 days of the study (May 11 to May 25).

Unlike DIN concentrations, dissolved organic nitrogen (DON) concentrations were relatively constant over the study period, ranging from 18.5 to 24.7 µM. Urea was <1% of the average bulk DON concentration. The highest DON concentrations were observed at the end of the study period following the dinoflagellate bloom.

Orthophosphate concentrations were low relative to DIN and ranged from below the analytical detection limit (0.03 µM) to 0.42 µM (Figure 6c). PO_4_^3−^ concentrations were lowest (at or near the detection limit) between 24 and 29 April, during the *Cryptomonas* spp. bloom and more variable during the *Gymnodinium* bloom.

Silicate concentrations were generally high averaging 30.6 µM (range 0.2–56.1 µM) (Figure 6d). However, over the period from 27 April to 8 May, during the *Cryptomonas* spp. bloom, silicate concentrations decreased from 31.7 to 0.2 µM. Following the precipitation on May 7 and May 8, silicate concentrations increased to 37.6 µM and remained high and increased during the remainder of the study. The ratio of dissolved silicate to DIN was greater than 16 except on May 8, suggesting that silicate concentrations were generally not limiting to diatom growth during the study period [20,48].

### 3.4. Time Lag Correlations

**Figure 7:**
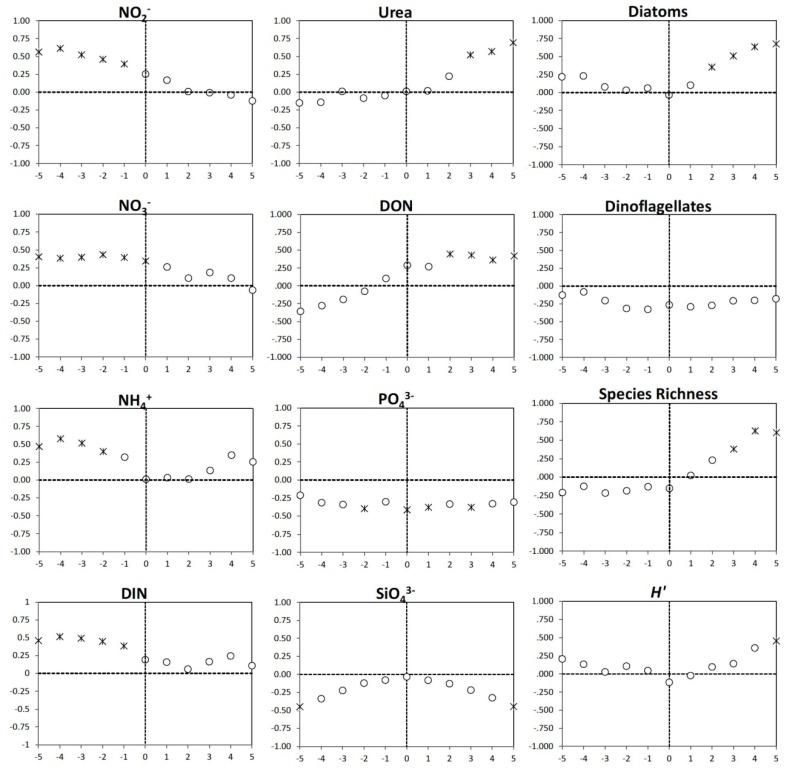
Time lag Pearson correlation plots of cryptomonad abundance versus nutrient concentrations, diatom abundance, dinoflagellate abundance, phytoplankton species richness and diversity (Shannon index *H′*). Periods of plus or minus five days are shown on the *X*-axis with 0 being present. The Pearson correlation coefficient is plotted on the *Y*-axis, with positive values indicating positive relationships, and negative values negative relationships. Correlations that are statistically significant at the *p* < 0.05 level are indicated by asterisks.

To understand the impact of environmental and biological conditions on the dominant phytoplankton in the community, time lagged correlations between cryptomonad and dinoflagellate abundances and individual nutrient concentrations and biological indicators were done. Significant positive correlations were observed between all forms of DIN and cryptomonad abundance 1 to 5 days later (Figure 7). These results suggest that with increases in DIN concentrations, cryptomonad populations respond by increasing their abundance. In contrast, the significant positive time lagged correlations between cryptomonad abundance and urea and DON concentrations indicate that urea and DON concentrations increased 2 to 5 days after cryptophyte abundance increased. This suggests that DON and urea were produced by or as a result of the cryptophyte bloom. There were negative correlations between PO_4_^3−^ and silicate concentrations and cryptomonad abundance both before and after the bloom. Cryptomonad abundance was also positively lag correlated with diatom abundance 2 to 5 days later, indicating that cryptomonad abundance might be related to the growth of diatoms, which could explain the negative correlation with silicate, albeit through an unknown mechanism Positive relationships between cryptomonad abundance and diversity were identified; significant correlations with species richness found 3 to 5 days after the bloom and *H′* five days after the bloom (Figure 7).

**Figure 8:**
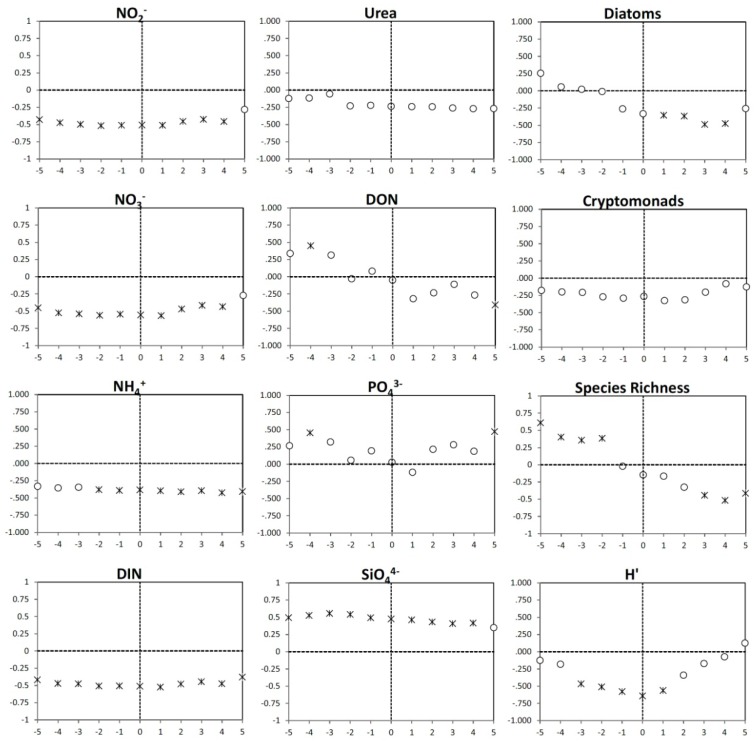
Time lag Pearson correlation plots of dinoflagellate abundance versus nutrient concentrations, diatom abundance, cryptomonad abundance, phytoplankton species richness and diversity (Shannon index *H′*). Periods of plus or minus five days are shown on the *X*-axis with 0 being present. The Pearson correlation coefficient is plotted on the *Y*-axis, with positive values indicating positive relationships, and negative values negative relationships. Correlations that are statistically significant at the *p* < 0.05 level are indicated by asterisks.

In contrast to the cryptomonads, dinoflagellates bloomed when DIN concentrations were at their lowest during the study period and as such, dinoflagellate abundance was negatively correlated with DIN concentrations, both in reverse and forward time (Figure 8). No significant correlations were found between urea concentrations and dinoflagellate abundance. Positive correlations between PO_4_^3−^ concentration and dinoflagellate abundance were identified, but significant only four days prior and five days after the bloom likely due to uptake by dinoflagellates during growth and regeneration after the bloom. Significant positive correlations were identified between silicate and dinoflagellate abundance, likely due to the fact that Si is not generally required for dinoflagellate growth and so was not being removed. Cryptomonad abundance and dinoflagellate abundance were negatively correlated, but not significantly (*p* < 0.05). Diatom abundance 1 to 4 days after the bloom was significantly negative correlated with dinoflagellate abundance; as dinoflagellate abundances decreased, diatom abundances increased. There were contrasting relationships identified between dinoflagellate abundance and diversity metrics. Dinoflagellate abundance was significantly positively correlated with species richness 2 to 5 days prior and negatively correlated 3 to 5 days after the bloom. Negative correlations between species diversity (*H′*) and dinoflagellate abundance were observed starting three days before the bloom and extending into the day after the bloom.

### 3.5. Discussion

Fundamental to understanding the distribution and abundance of phytoplankton groups is their relationship to environmental variables that vary over short and long timescales in estuarine environments such as the Chesapeake Bay [49,50]. Estuaries are dynamic environments where chemical and physical parameters can vary over short time periods (e.g., tidal, sub-tidal, and diurnal), in response to episodic events such as storms (e.g., heavy rainfall and wind that impact salinity, temperature, stratification, turbidity, and nutrient concentrations), and over longer time scales due to climatic and anthropogenic forcing [21,51,52,53,54]. This study was aimed at understanding how environmental and biological factors combine to favor the formation of monospecific algal blooms over relatively short timescales during spring when rainfall and air and water temperatures can be highly variable resulting in short-term changes in salinity and nutrient concentrations in surface waters.

During the course of this 34-day study, two distinct blooms developed and dissipated, each over an approximately 7-day period. The first was a due to cryptomonad species common to the Lafayette River estuary, and the second a dinoflagellate, *Gymnodinium instriatum*. Neither bloom was detected during monthly monitoring of the estuary. Cryptomonads are a common component of estuarine phytoplankton communities throughout the year and comprise a major portion of the algal biomass in Virginia estuaries [55]. Their abundance has been associated with disturbances such as wind induced mixing of the water column and precipitation [44,56]. Cryptomonads are also readily preyed on by grazers that include ciliates, cladocerans, copepods, and dinoflagellates [44,57,58]. *Gymnodinium instriatum* (Freudenthal et Lee) Coats is an unarmored dinoflagellate that can form dense blooms, often producing “red tides” in coastal waters throughout the world, and has been associated with shellfish mortality through oxygen depletion [5,6,7,8,9,10,11,12,13,14,15,16,17,18,19,20,21,22,23,24,25,26,27,28,29,30,31,32,33,34,35,36,37,38,39,40,41,42,43,44,45,46,47,48,49,50,51,52,53,54,55,56,57,58,59,60]. *G. instriatum* is mixotrophic, and has been reported to feed on a variety of ciliates [61]. It is likely that it can also feed on cryptophytes, as has been observed for other mixotrophic dinoflagellates [62,63] *G. instriatum*, like many dinoflagellates is also capable of forming cysts when under environmental stress [64]. While this species has a wide salinity tolerance and is a common component of the phytoplankton community in tropical and temperate estuaries [46,47,48,49,50,51,52,53,54,55,56,57,58,59,60,61,62,63,64,65], its abundance in the Chesapeake Bay estuary is largely unknown due to its gross morphological similarity to a variety of other *Gymnodinium* and *Gyrodinium* dinoflagellates. It has been documented within the Bay using molecular techniques [47,66]; however, little is known about the conditions promoting blooms of *G. instriatum* in the environment [65].

Seasonal changes in water temperature and water quality affect species succession, the composition of plankton communities, and the particular organisms available to bloom seasonally [17,67,68,69]. During bloom initiation, the concentrations of particular algal species or assemblages within the planktonic community can change rapidly, often resulting in dominance by a single or a few species and reduced algal species diversity [4]. Monospecific blooms can develop and deteriorate over short time periods or may extend for months in estuarine systems [21,22].

One short-term forcing function that impacts physical and chemical conditions in temperate estuaries is storms and their associated wind and rainfall [52,70,71]. In shallow systems high winds can inject nutrients from the sediments as a result of intense mixing. In addition, rainfall can deliver nutrients directly through wet deposition or indirectly through overland and stream-flow. Inputs of freshwater can enhance stratification through the introduction of buoyancy. In many estuarine environments, wetlands and aquatic shoreline vegetation buffer the effects of seasonal or sporadic runoff by removing nutrients before they enter the estuary [72,73,74]. However, urban environments such as the Lafayette River, where the shoreline is highly developed and marsh covers less than half of its shoreline, storm water can enter the estuary directly through overland flow which is facilitated by impervious surfaces [29]. As a result, even relatively brief precipitation events can lead to large and rapid changes in water quality from storm sewer discharge and overland runoff [51,52,53,54,55,56,57,58,59,60,61,62,63,64,65,66,67,68,69,70,71,72,73,74,75].

Increases in *Cryptomonas* spp. cell density in the Lafayette River were first detected 48 h after a storm that delivered 0.74 cm of rain on 22 April. Cell densities reached their maximum about 48 h after a second rainfall of 2.8 cm on 25 April. The rainfall resulted in a decrease in surface salinity and increases in dissolved inorganic nitrogen concentrations, particularly NO_3_^+^ and NH_4_^+^. While the densities of *Cryptomonas* spp. increased rapidly, those of diatoms and other phytoplankton decreased. This resulted in a lower Shannon diversity index (*H′*). There was no corresponding decline in species richness, indicating the reduction in diversity was due to reduced species evenness. During the height of this bloom, *Cryptomonas* spp. dominated the phytoplankton community, comprising 91% of the algal biomass (Figure 2b). Based on daily changes in their abundance, the apparent net growth rate of *Cryptomonas* sp. during the period between 22 and 27 April was 0.86 divisions per day, similar to upper limits of *Cryptomonas* growth rates observed in cultures [76]. Because this estimate does not take into account potential losses due to grazing or advection, this rate should be considered a conservative one.

While ammonium concentrations increased after rainfall events, concentrations decreased concomitantly with increases in *Cryptomonas* abundance, suggesting its rapid uptake to support cellular growth. This is consistent with laboratory studies demonstrating high rates of ammonium uptake by *Cryptomonas* [77]. Within Chesapeake Bay, ammonium can be the dominant N form taken up by phytoplankton [78] and is often higher during summer as a result of N recycling [79]. Ammonium and nitrate levels increased again following rainfall on May 7–8. Again, ammonium concentrations declined more rapidly than nitrate, and this coincided with another peak in *Cryptomonas* abundance. DIN concentrations and *Cryptomonas* abundance were positively related in the lag-correlation analyses at periods of 1–5 days (Figure 7) suggesting that nutrient inputs from runoff may have stimulated *Cryptomonas* growth. 

*Gymnodinium instriatum* was present at low cell densities (<100 cells mL^−1^) for the first 25 days of the study. However, about 48 h following the rainfall on May 14 and 15, *G. instriatum* populations burgeoned to over 30,000 cells mL^−1^, an apparent net growth rate of 3.3 divisions per day. This was over four times greater than the maximum growth rate reported for this species in laboratory cultures [65] but again did not account for grazing and advection losses or the effects of dinoflagellate swarming and aggregation [80]. A synchronous excystment of benthic dinocysts from river sediment may also have contributed to this rapid increase in *G. instriatum* abundance. Shikata *et al*. [64] (2008) showed that *G. instriatum* can excyst over a short period of time (≤3 days) at water temperatures ≥20 °C, consistent with those present during the time this bloom initiated.

Dinoflagellate cyst-beds are produced by several estuarine dinoflagellate species, and are thought to serve as a survival mechanism for organisms living in habitats where environmental conditions fluctuate [81,82]. Cyst formation in *G. instriatum* has been attributed to N and P limitation [64] and high cell densities [61]. High densities of a variety of benthic dinoflagellate cysts have been identified in tributaries of the lower Chesapeake Bay, including the Elizabeth and Lafayette Rivers [39,81]. Increases in summertime blooms of the pelagic dinoflagellate *Cochlodinium polykrikoides* in the Lafayette River and elsewhere in the lower Chesapeake Bay estuary have also been attributed to local cyst-beds [83,84]. Excystment of *C. polykrikoides* appears to be triggered by following summer rainfall events when water temperatures are ≥26 °C, likely due to nutrient inputs and enhanced stratification [7,21,22]. Water temperatures just prior to the rainfall May 14 and 15 had reached 22.6 °C. Following rainfall and during the subsequent *G. instriatum* bloom, DIN concentrations were near or below the analytical detection limit, likely because it was rapidly taken up by the emerging and rapidly growing dinoflagellates and so never accumulated in the environment.

While excystment and population growth of *G. instriatum* may be stimulated by runoff of nutrients into the river, this is not strongly supported by the lag correlation analysis. Instead, the opposite pattern was observed, with DIN concentrations negatively correlated with dinoflagellate abundance. Many harmful algal bloom taxa, including dinoflagellates, occur during periods when nutrients, particularly DIN, are depleted [17,21,22,85]. These conditions are thought to favor blooms of mixotrophic dinoflagellates over other taxa such as diatoms and cryptophytes that are more autotrophic [85]. Many dinoflagellates can use dissolved organic N (DON) and graze on co-occurring microbes including cryptophytes and unicellular cyanobacteria to augment their nutrition [86,87].

The *G. instriatum* bloom appeared to initiate after a spring storm and in addition to the associated nutrient loading, freshwater inputs also enhance stratification which can lead to rapid increases in dinoflagellate abundance relative to diatoms, including through excystment [21,88,89,90]. Alternatively, the high apparent net growth rate observed at the sampling location may be attributed to advection and transport of cells from elsewhere in the estuary. Collections were made at the same tidal period to reduce the impact of cyclical tidal advection on the observations, however freshwater input related flushing still would have an effect on transporting water mass and the plankton therein. Algal blooms are spatially heterogeneous and patchy, and it is possible that a bloom had developed upstream in response to nitrogen loading elsewhere, before being transported downstream through flushing. In this scenario, the dinoflagellate bloom may also have been directly related to increased nutrient inputs upstream, with depleted DIN concentrations occurring during transport before the bloom was sampled at the study site [91]. Uncertainty related to the origin of the bloom is an inherent property of a study of a dynamic water body with limited spatial coverage.

Additionally, species interactions, such as the abundance of potential algal prey, could have stimulated *G. instriatum* growth as have been shown for other mixotrophic dinoflagellates. Toxic *Karlodinium veneficum* blooms have been correlated with increases in cryptophyte abundance through the stimulation of grazing [58]. While grazing by *G. instriatum* was not measured in this study, *Cryptomonas* sp. abundances decreased as *G. instriatum* concentrations increased, and cryptophyte concentrations were the lowest during the dinoflagellate bloom (Figure 4b); however, cryptomonad and dinoflagellate abundance were not significantly correlated (Figure 7 and Figure 8).

Algal diversity (*H′*) was greatly reduced during both blooms, primarily due to decreased species evenness. This led to the significant negative correlation between species diversity and algal biomass (Figure 5a). Examination of diversity/productivity relationships in both terrestrial and aquatic systems have identified positive, negative and unimodal associations [41,92]. Similar studies of phytoplankton communities are more limited; however it appears that at a large enough productivity gradient, the relationship appears to be unimodal, with maximum diversity at intermediate phytoplankton biomass concentrations [93]. Surprisingly, while species diversity was low during blooms due to low evenness, phytoplankton species richness remained relatively high during blooms suggesting community resilience.

The phytoplankton abundances observed in this study were as much as an order of magnitude greater than those observed at Chesapeake Bay Program monthly monitoring stations in the polyhaline lower Chesapeake Bay watershed at the same time period [94]. Phytoplankton abundances observed during this study during the incoming tide (about 2 h after low tide) at our shallow-water station in the Lafayette River (5.7 × 10^6^–7.8 × 10^7^ cells L^−1^) were higher than those recorded at monthly monitoring stations occupied as part of the Virginia Chesapeake Bay Monitoring Program (CBMP) (1.8 × 10^6^–1.3 × 10^7^ cells L^−1^) during the same time period [94]. This highlights the unlikelihood of capturing blooms during monthly monitoring and the need for developing different tools for assessing these ephemeral events so that we can begin to understand causal factors promoting blooms in the environment.

## 4. Conclusions

Dinoflagellate blooms, appear to be increasing in magnitude and frequency in Chesapeake Bay and its tributaries and this has been linked to nutrient loading and eutrophication [20,23,34,95]. We examined the effects of water quality on phytoplankton community composition in an urban estuarine tributary susceptible to storm water input and seasonal dinoflagellate blooms. Blooms are ephemeral in nature and can develop rapidly in response to environmental forcing and so are undersampled by routine monthly monitoring programs. During this study, blooms of *Cryptomonas* sp. and *G. instriatum* developed and dispersed over short periods (5–7 days) of time between storms. Blooms were associated with reduced algal diversity but species richness remained relatively constant during the blooms. The rapid development and brief duration of both blooms emphasizes the importance of monitoring on appropriate temporal scales. The study also illustrates the compromises that are required when designing a monitoring experiment, as increased spatial coverage of the tributary may have helped explain bloom initiation and advection, but at the expense of temporal periodicity. These results build on a growing number of studies that indicate bloom emergence is complex, with water quality parameters (e.g., nutrient loading), hydrologic transport, and species interactions all contributing to bloom formation by mixotrophic taxa.
